# Supplementary material for: A functional parcellation of the whole brain in high-functioning individuals with autism spectrum disorder reveals atypical patterns of network organization
Source: Mol Psychiatry. 2024 Sep 30;30(4):1518–28. doi: 10.1038/s41380-024-02764-6 (PMC11919759; doi:10.1038/s41380-024-02764-6)
Supplement: Supplementary file 1 — Supplemental Material [file 41380_2024_2764_MOESM1_ESM.pdf]

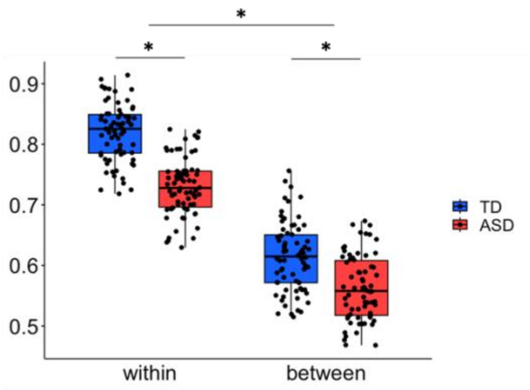

Supp. Figure 1. The Δ eta^2 coefficient separated into within and between network voxels and then averaged across the five networks that showed a significant group difference in Figure 4. A significant Group x Within/Between interaction ($p<0.0001$) shows a greater decrease in within-network eta^2 coefficients compared to between-network eta^2 coefficients in these regions of the ASD group.
